# Preventable clinical and psychosocial factors predicted two out of three recurrent cardiovascular events in a coronary population

**DOI:** 10.1186/s12872-020-01368-6

**Published:** 2020-02-05

**Authors:** E. Sverre, K. Peersen, H. Weedon-Fekjær, J. Perk, E. Gjertsen, E. Husebye, L. Gullestad, T. Dammen, J. E. Otterstad, J. Munkhaugen

**Affiliations:** 1 Department of Medicine, Drammen Hospital, Drammen, Norway; 2 Department of Behavioural Sciences in Medicine and Faculty of Medicine, University of Oslo, Oslo, Norway; 3 Department of Medicine, Vestfold Hospital, Oslo, Norway; 4 Oslo Centre for Biostatistics and Epidemiology, Research Support Services, Oslo University Hospital, Oslo, Norway; 5 Department of Cardiology, Public Health Department Linnaeus University, Kalmar, Sweden; 6 Department of Cardiology, Oslo University Hospital Rikshospitalet, Faculty of Medicine, University of Oslo, Oslo, Norway; 7 KG Jebsen Cardiac Research Center, Oslo University Hospital Ullevål, Oslo, Norway

**Keywords:** Coronary heart disease, Secondary prevention, Risk factors, Psychosocial factors, Prognosis, Recurrent cardiovascular events

## Abstract

**Background:**

The relative importance of lifestyle, medical and psychosocial factors on the risk of recurrent major cardiovascular (CV) events (MACE) in coronary patients’ needs to be identified. The main objective of this study is to estimate the association between potentially preventable factors on MACE in an outpatient coronary population from routine clinical practice.

**Methods:**

This prospective follow-up study of recurrent MACE, determine the predictive impact of risk factors and a wide range of relevant co-factors recorded at baseline. The baseline study included 1127 consecutive patients 2–36 months after myocardial infarction (MI) and/or revascularization procedure. The primary composite endpoint of recurrent MACE defined as CV death, hospitalization due to MI, revascularization, stroke/transitory ischemic attacks or heart failure was obtained from hospital records. Data were analysed using cox proportional hazard regression, stratified by prior coronary events before the index event.

**Results:**

During a mean follow-up of 4.2 years from study inclusion (mean time from index event to end of study 5.7 years), 364 MACE occurred in 240 patients (21, 95% confidence interval: 19 to 24%), of which 39 were CV deaths. In multi-adjusted analyses, the strongest predictor of MACE was not taking statins (Relative risk [RR] 2.13), succeeded by physical inactivity (RR 1.73), peripheral artery disease (RR 1.73), chronic kidney failure (RR 1.52), former smoking (RR 1.46) and higher Hospital Anxiety and Depression Scale-Depression subscale score (RR 1.04 per unit increase). Preventable and potentially modifiable factors addressed accounted for 66% (95% confidence interval: 49 to 77%) of the risk for recurrent events. The major contributions were smoking, low physical activity, not taking statins, not participating in cardiac rehabilitation and diabetes.

**Conclusions:**

Coronary patients were at high risk of recurrent MACE. Potentially preventable clinical and psychosocial factors predicted two out of three MACE, which is why these factors should be targeted in coronary populations.

**Trial registration:**

Registered at ClinicalTrials.gov: NCT02309255.

Registered at December 5th, 2014, registered retrospectively.

## Background

Improved treatment of acute coronary syndrome with revascularization and modern medical drug therapy has reduced the short-term mortality rates and increased the number of coronary heart disease (CHD) patients in need of secondary prevention [1]. In most European countries, primary care physicians are the key actors to coordinate and provide long-term CHD management [1]. Efforts to support their clinical work is needed, as data from clinical practice in Europe have revealed poor risk factor control [2] with only few improvements over time [3]. Unhealthy lifestyle behaviour and low risk factor control is shown to contribute to the high risk of recurrent cardiovascular (CV) events observed in CHD patients [4, 5].

The relative importance of different determinants of long-term disease progression need to be studied further, since most previous studies are based on registries [4–6] with a limited number of clinical factors included. Data on lifestyle behaviour, participation in cardiac rehabilitation (CR) programs, and psychosocial factors have frequently been missing. In the recent EuroAspire IV registry study, CV comorbidities, low education and depressive symptoms were strongly and significantly associated with CV death or non-fatal myocardial infarction (MI), stroke or heart failure in an outpatient population, whereas lifestyle factors and control of lipids and blood pressure (BP) were not [7].

The current prospective study aims to estimate the relative importance of preventable and potentially modifiable clinical and psychosocial factors associated with recurrent major adverse CV events (MACE) in an outpatient coronary population from routine clinical practice.

## Methods

### Design and study population

**Fig. 1:**
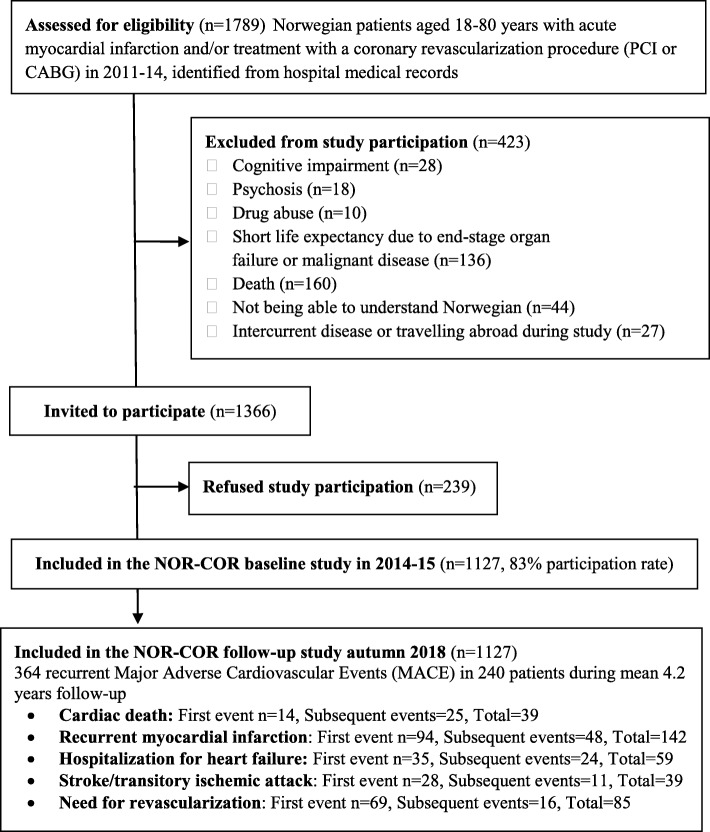
Study flow chart

This prospective cohort study is part of the larger NORwegian CORonary (NOR-COR) prevention project [8] (Fig. 1 - *study flow chart*). We identified 1789 consecutive patients aged 18–80 years with a first or recurrent coronary event in 2011–2014 from the catchment areas of the Norwegian hospitals in Drammen and Vestfold. The participation rate was 83% after excluding 423 patients with failing eligibility and omitting 239 patients who refused participation. The remaining 1127 patients were included during 2014–2015, with a median time of 16 months (range 2–36) after the coronary index event. In patients with a history of several coronary events prior to study inclusion, the last event was defined as the index event. Participants answered a comprehensive questionnaire and underwent a clinical examination with blood sample collections. Data on recurrent CV events after baseline were collected from the patients` hospital medical records in 2018, after a mean follow-up of 4.2 years. Complete follow-up data were missing in only 14 (1.2%) patients who had moved out of the catchment area of the participating hospitals since study inclusion.

The two participating hospitals have a catchment area of 380,000 inhabitants corresponding to 7.4% of the Norwegian population. The catchment area has a representative blend of city and rural districts and is representative of Norwegian education, economy, age distribution, morbidity, and mortality [9, 10]. The CR programs differ in content and availability between the two participating hospitals [11]. At Drammen Hospital, CR includes a multi-disciplinary 1 day “heart school”, and exercise training twice per week for 6 weeks. The Hospital of Vestfold provides more comprehensive lifestyle intervention lasting for up to 6 months [11].

### Ethics, consent and permission

The NOR-COR study was approved by the Regional Committee of Ethics in Medical Research (2013/1885). All patients signed a written informed consent prior to study participation.

### Outcome assessment

The primary predefined [8] composite endpoint of recurrent MACE comprising CV death or readmission for myocardial infarction (MI), new revascularization procedure (PCI or CABG) due to stable/unstable angina, stroke/transitory ischemic attacks (TIA) or heart failure was obtained from the hospital records between October 10th and November 30th 2018. The registration was performed by two experienced cardiac researchers, as medical diagnoses obtained from hospital medical records are often regarded the gold standard [12].

### Registered study variables

Covariates registered at baseline (2014–15, 8]:

- From hospital medical records: Age, sex, coronary history and treatment, CV comorbidity and participation in CR.
- From questionnaire: Education, smoking history including years smoked, physical activity, CV medication, self-reported family history of premature CHD (< 55 years male and < 65 years females) in first degree relatives, adherence and anxiety and depression symptoms (Hospital Anxiety and Depression Scale (HADS)).
- From blood samples: Total cholesterol, low-density lipoprotein (LDL) cholesterol, high-density lipoprotein (HDL) cholesterol and C-reactive protein (CRP) (Architect ci16200, Abbott Laboratories, USA), and HbA1c (Tosoh G8, Tosoh Medics Inc., USA). All blood samples were analysed at Drammen hospital to avoid interlaboratory bias.
- From clinical examination: Waist circumference (nearest 0.5 cm), height (nearest 0.5 cm) and weight (nearest 0.5 kg). Systolic and diastolic BP were measured with standardized procedure using a validated digital sphygmomanometer (Welch Allyn Connex ProBP 3400).

### Statistical analyses

The descriptive baseline measurements are presented as frequencies and percentages for proportions, and as mean with standard deviation (SD) for continuous variables. Differences between groups were tested by χ^2^ tests and *t-*tests. Cox proportional hazard models were used to calculate relative risk (RR) and 95% confidence interval (CI) for first and MACE event after study inclusion. Analysis time in the Cox model was defined by the time from the index event, in practice adjusting for all baseline variations in risk by time since the previous (index) coronary event (using left-truncated data with censoring). Patients were followed until the date of a recurrent event or the end of study (1st December 2018), whichever occurred first. Data were also analysed using all MACE events, to evaluate whether results were consistent with the increased number of end-points and a more biologically mixed dataset. We first identified the relevant non-modifiable and modifiable covariates a priori, and adjusted for these in the multivariable Cox regression analyses. Since patients with established CHD prior to inclusion were presumed to have different risk level and profile by study time, all analyses were stratified for prior CHD before the index event.

Most applied variables had few missing values (range: 0–10%). However, in the multivariable Cox regression analysis the combination of missing values for different variables resulted in 290 excluded patients (including 58 patients with a MACE). These missing cases lowered the statistical power of the study and could potentially have introduced a systematic bias. Hence, we also performed multivariate regression imputation under a missing at random assumption [13].

While relative risk is a good measurement of the observed risk differences across co-variables, the population impact of a given co-variable also depends on frequency of the co-variable. Hence, we also estimated the population attributable fraction (PAF) for each factor(s), measuring the factor(s) estimated contribution to the overall expected risk of MACE events [14]. For a true modifiable risk factor, this population attributable fraction equals the estimated proportion of cases that could be prevented by changing the given factor. As the effect of each co-variate in the Cox model is multiplicative, the combined PAF will be smaller than the sum of the individual PAF’s, highlighting the lower potential effect of prevention when the overall risk decreases. As our PAF analyses takes the prevalence in the given population into account, it yields an estimate for the clinical significance of the given risk factor in our outpatient coronary population. Statistical analyses were performed using Stata version 15 (StataCorp LLC, College Station, USA), with the PAF calculated by the punafcc Stata add-on package [14].

## Results

Mean age at study inclusion was 63.6 (SD 9.6) years and 21% were females *(*Table 1*)*. The index coronary event was MI in 80% and stable or unstable CHD with angiography-verified stenosis in 20%. In all, 90% had been revascularized, 97% used at least one antiplatelet agent, 92% used a statin and 47% had participated in CR. Thirty percent (*n* = 336) had coronary event(s) prior to the index event. Thirty-four percent were obese (BMI > 30 kg/m^2^), 21% were current smokers, and 54% were former smokers. In all, 96% of the current smokers and 75% of the former smokers had been smoking for ≥20 years.

**Table 1:** Baseline characteristics for the study population

	All patients (*n* = 1127)
Mean age at inclusion, mean ± SD	63.6 ± 9.6
Females, n (%)	237 (21.0)
Low education^a^, n (%)	780 (70.3)
Family history of coronary heart disease^b^, n (%)	481 (42.5)
ST-elevation infarction, n (%)	335 (29.7)
Non-ST-elevation infarction, n (%)	561 (49.8)
Stable or unstable angina, n (%)	231 (20.4)
Percutaneous coronary intervention, n (%)	867 (77.3)
Coronary artery bypass graft operation n (%)	147 (9.6)
No revascularization, n (%)	108 (9.6)
≥ 1 coronary event prior to index event, n (%)	336 (29.8)
Heart failure, n (%)	148 (13.1)
Atrial fibrillation, n (%)	106 (9.4)
Peripheral artery disease, n (%)	100 (8.9)
Stroke or transient ischemic attack, n (%)	80 (7.1)
Chronic kidney failure (eGFR< 60 mL/min/1.73m^2^), n (%)	139 (13.4)
Participation in cardiac rehabilitation, n (%)	526 (46.7)
Former smoking, n (%)	603 (55.7)
Current smoking, n (%)	230 (21.2)
Total cholesterol, mean ± SD	4.0 ± 1.0
Low density lipoprotein cholesterol, mean ± SD	2.1 ± 0.8
High density lipoprotein cholesterol, mean ± SD	1.1 ± 0.3
Non-high density lipoprotein cholesterol, mean ± SD	2.9 ± 0.9
Low physical activity^c^, n (%)	472 (41.9)
Physical inactivity^c^ n (%)	197 (18.0)
Diabetes mellitus, n (%)	189 (16.9)
HbA1c in non-diabetic patients, mean ± SD	5.8 ± 0.5
HbA1c in diabetic patients, mean ± SD	7.6 ± 1.4
Systolic blood pressure mmHg, mean ± SD	138 ± 19.0
Diastolic blood pressure mmHg, mean ± SD	82 ± 8.8
Waist circumference cm, mean ± SD	102.5 ± 12.3
Body Mass index in kg/m^2^, mean ± SD	28.6 ± 4.5
C-reactive protein mg/l, mean ± SD	2.5 ± 2.7
At least 1 antiplatelet agent, n (%)	1096 (97.2)
Statin treatment, n (%)	1036 (91.9)
Beta-blocker treatment, n (%)	815 (72.3)
ACE inhibitor or ARB treatment, n (%)	561 (49.8)
HADS Anxiety sum score, mean ± SD	4.8 ± 3.2
HADS Depression sum score, mean ± SD	3.9 ± 3.2

*SD* Standard deviation, *HADS* Hospital Anxiety and Depression score, *eGFR* estimated glomerular filtration rate, *ACE* Angiotensin converting enzyme, *ARB* Angiotensin receptor blocker

^a^Completion of primary or secondary school only

^b^Family history of coronary heart disease was defined as first degree relatives with coronary heart disease before the age of 55 years for men and 65 years for women

^c^Adequate physical activity is defined as ≥moderate physical activity for 30 min 2–3 times a week, low physical activity is defined as <moderate physical activity for 30 min 2–3 times a week, and physical inactivity as physical activity < 1 time a week

During a mean follow-up period of 4.2 (SD 0.4) years after study inclusion (mean time from index event to end of study was 5.7 (SD 0.9) years), 364 MACE events were observed in 240 (21 95% CI, 19–24%) patients, whereas 39 (3.4, 0.8% per year) died of CV causes. The distribution of composite endpoints is provided in Fig. 1*.* The risk of recurrent MACE was significantly higher in patients with CHD prior to the index event compared to those without (age adjusted RR = 2.37, 95% CI 1.84–3.07, *p* < 0.001).

The long-term risk of first MACE was significantly associated with increasing age, low education, former smoking, peripheral artery disease (PAD), chronic kidney failure and prior stroke, but not with gender in analyses adjusted for age and stratified by coronary events prior the index event *(*Table 2*, model 1).* Of the potentially modifiable risk factors not taking statins, low or no physical activity, diabetes, non-participation in CR, higher systolic BP and higher anxiety and depression scores (HADS) were significantly associated with MACE. Current smoking vs. never smoking (RR 1.24, 95% CI 1.01–1.53, *p* = 0.048) was also associated with MACE (data not shown). In multi-adjusted analyses (Table 2*model 3*), the strongest potentially modifiable predictors of MACE were not taking statins, physical inactivity and higher depression scores. Diabetes, non-participation in CR and higher anxiety score (HADS) were significantly associated with MACE after adjustments for coronary risk factors (Table 2*, model 2*), but became borderline significant after additional adjustments for CV comorbidity. In multi-adjusted sub-group analyses in patients with no CHD prior to the index event (*n* = 791), LDL-cholesterol (RR 1.38 per mmol/L increase, 95% CI 1.13–1.68, *p* = 0.002) was significantly associated with MACE. As no major changes in the estimates of the potentially modifiable factors were observed, study results for all MACE are presented in Additional file 1.

**Table 2:** Risk of first recurrent cardiovascular event in coronary patients, estimated by Cox proportional hazard regression

	Model 1^a^		Model 2^b^		Modell 3^c^	
	RR	*p*-value	RR	*p*-value	RR	*p*-value
Age per 10 years	1.15 (1.00, 1.32)	0.050	1.12 (0.96, 1.30)	0.146	1.02 (0.87, 1.20)	0.792
Male sex	0.85 (0.63, 1.15)	0.299	0.93 (0.67, 1.27)	0.638	0.89 (0.64, 1.23)	0.472
Low education^d^	1.68 (1.23, 2.31)	0.001	1.58 (1.15, 2.11)	0.005	1.51 (1.09, 2.09)	0.014
Never smoking	1 (reference)		1 (reference)		1 (reference)	
Former smoking	1.54 (1.09, 2.19)	0.017	1.51 (1.06, 2.16)	0.024	1.46 (1.01, 2.10)	0.043
Current smoking	1.48 (0.97, 2.24)	0.067	1.32 (0.86, 2.02)	0.205	1.13 (0.73, 1.76)	0.587
Adequate physical activity^e^	1 (reference)		1 (reference)		1 (reference)	
Low physical activity	1.36 (1.00, 1.85)	0.051	1.37 (1.00, 1.87)	0.049	1.35 (0.97, 1.87)	0.071
Physical inactivity	1.84 (1.29, 2.61)	0.001	1.78 (1.23, 2.58)	0.002	1.73 (1.18, 2.55)	0.005
LDL cholesterol per mmol/L increase	1.17 (1.00, 1.38)	0.057	1.17 (0.99, 1.37)	0.063	1.14 (0.97, 1.35)	0.119
Diabetes mellitus	1.64 (1.22, 2.19)	0.001	1.47 (1.09, 2.00)	0.013	1.35 (0.99, 1.84)	0.061
Systolic blood pressure per 10 mmHg increase	1.06 (0.99, 1.13)	0.111	1.06 (0.99, 1.13)	0.088	1.06 (0.99, 1.13)	0.114
Waist circumference per 10 cm increase	1.24 (1.00, 1.54)	0.048	1.06 (0.84, 1.33)	0.639	1.05 (0.83, 1.33)	0.687
C-reactive protein per mg/L increase	1.02 (0.98, 1.06)	0.346				
Not participating in cardiac rehabilitation	1.42 (1.09, 1.86)	0.010	1.32 (1.01, 1.74)	0.045	1.29 (0.97, 1.70)	0.077
Not taking statin	2.08 (1.43, 3.03)	< 0.001	2.06 (1.33, 3.20)	0.001	2.13 (1.36, 3.36)	0.001
Heart failure	1.21 (0.86, 1.69)	0.281				
Peripheral artery disease	1.96 (1.39, 2.75)	< 0.001	1.78 (1.26, 2.52)	0.001	1.73 (1.21, 2.49)	0.003
Stroke or transient ischemic attack	1.43 (0.94, 2.16)	0.091	1.28 (0.84, 1.95)	0.248	1.12 (0.72, 1.74)	0.617
Chronic kidney failure (eGFR< 60 mL/min/1.73m^2^)	1.84 (1.32,2.35)	< 0.001	1.62 (1.16, 2.27)	0.005	1.52 (1.08, 2.14)	0.016
HADS Anxiety sum per unit increase	1.04 (1.01, 1.08)	0.017	1.03 (1.00, 1.07)	0.031	1.03 (1.00, 1.07)	0.058
HADS Depression sum per unit increase	1.06 (1.02, 1.10)	0.002	1.05 (1.01, 1.09)	0.045	1.04 (1.00, 1.09)	0.028

*RR* Relative risk, *LDL* Low density lipoprotein cholesterol, *eGFR* estimated glomerular filtration rate, *HADS* Hospital anxiety and depression score

All analyses based on imputed dataset

^a^Adjusted for age. Analysis is stratified by prior coronary events before the index event or not

^b^Adjusted for coronary risk factors with p-value< 0.1 in crude or age adjusted analyses (smoking, LDL cholesterol, physical activity and systolic blood pressure) in addition to adjustments in Model 1

^c^Adjusted for cardiovascular comorbidity with *p*-value < 0.1 in crude analyses (stroke, peripheral artery disease and kidney failure) in addition to adjustments in Model 2

^d^Completion of primary or secondary school only

^e^Adequate physical activity is defined as ≥moderate physical activity for 30 min 2–3 times a week, low physical activity is defined as <moderate physical activity for 30 min 2–3 times a week, and physical inactivity as physical activity < 1 time a week

The preventable and the potentially modifiable risk factors accounted for 66% (95% CI 49–77%) of the risk of MACE in population attributable fractions (PAF) analyses stratified for prior CHD at the index event and adjusted for age (Table 3). History of smoking (current and former) gave the highest contribution (27%), followed by low physical activity, not participating in CR (16%), diabetes (7%) and not taking statins (7%). By adding CV comorbidity, the PAF for all factors increased by only 2 to 68%. The PAF for all factors did not change after excluding patients with CV comorbidity (data not shown).

**Table 3:** Attributable risk fraction associated with preventable and potentially modifiable risk factors

Attributable risk fraction (95% confidence interval)		
History of smoking	27%	(5, 44)
LDL cholesterol ≥1.8 mmol/L	−4%	(−23, 12)
Low physical activity^a^	21%	(5, 34)
Diabetes mellitus	7%	(1, 13)
Blood pressure ≥ 140/90 (80) mmHg	7%	(−6,19)
Central obesity^b^	11%	(−8, 28)
Not participating in cardiac rehabilitation	16%	(1, 28)
Not taking statin	7%	(4, 9)
HADS Anxiety or Depression score ≥ 8	7%	(0, 15)
All risk factors combined	66%	(49, 77)

*LDL* Low density lipoprotein, *HADS* Hospital anxiety and depression scale

Analyses based on imputed dataset

^a^Less than 30 min of moderate activity 2–3 times a week

^b^Waist circumference ≥ 102 cm in males and ≥ 88 cm in females

## Discussion

The risk of recurrent non-fatal CV events remained high in a chronic outpatient coronary population from routine clinical practice in Norway. Not taking statins, low or no physical activity and higher depression scores were the major potentially modifiable risk factors associated with MACE in multi-adjusted analysis. The comprehensive NOR-COR dataset, enables us to determine the relative importance of preventable and potentially modifiable factors that are regularly assessed in daily practice. Altogether, potentially preventable clinical and psychosocial factors predicted two out of three MACE in the present study. This emphasizes the great potential for reducing the patients` long-term residual CV mortality and morbidity risk by optimizing these factors.

The study population was < 80 years and most patients were revascularized and received the recommended drug treatment which is subsidized in Norway. Despite this, more than 2 out of 10 patients suffered a MACE during a 4 years follow-up period, and 16% had a non-fatal MI, stroke or CV death giving a yearly rate of 3.8%. Our results are in line with older data from the REACH registry reporting a prevalence of recurrent first CV death, MI and stroke of 4.5% per year (18% over 4 years) in patients with established CVD. In contrast, the EuroAspire IV register, with similar inclusion criteria, found a yearly rate of first CV death, MI and stroke of only 2.6% (5.1% over 2 years). However, EuroAspire IV had an inclusion rate of only 49% [7] whereas 60% of the MACE were obtained by self-rapport questionnaires which may have underestimated the true prevalence. In line with our results, there was a yearly incidence of 1.1% CV deaths in EuroAspire IV. The high levels of MACE along with a low incidence of CV deaths found in both studies, most probably reflect effective management of recurrent non-fatal MACE.

Current smoking was not significantly associated with MACE compared to former and never smoking although a trend towards increased risk was observed. Former smoking, however, was prevalent and significantly associated with MACE. Even though former smoking may be regarded as a non-modifiable factor, it is a preventable risk factor in the CHD population. Smoking history combining former and current smoking, accounted for the highest attributable risk fraction (27%) for recurrent MACE. In line with our results, EuroAspire IV [7], did not find a significant association between current smoking and MACE. Possible explanations might be a long history of smoking in those quitting prior to study inclusion and too short follow-up to see the effect of smoking cessation. The susceptibility of smoking also differs individually [14], and those patients most susceptible to the negative effects of smoking, might to a larger extent have died prior to study inclusion. Smoking was significantly associated with increased risk of death, but not readmissions in a large Swedish registry study [4]. Nevertheless, the benefit of smoking cessation in CHD prevention is strongly documented [15].

Physical inactivity (< 1 time/week) was a strong predictor of MACE. Low physical activity (< 30 min 2–3 times/week) was also associated with MACE after adjusting for other CV risk factors, but the association became borderline significant (*p* = 0.071) after adjusting for CV comorbidity. The EuroAspire IV study did not find low physical activity to be significantly associated with MACE, but low physical activity was defined differently [7]. However, several observational studies have identified physical inactivity as an important prognostic factor in CHD patients [4, 16]. Other studies have found that the greatest effect on CHD prognosis was achieved by increasing the activity level from inactivity to low activity [17, 18]. Therefore a larger effort should be made to help inactive patients become somewhat active, even though they may not reach guideline recommendations [1].

A recent review found an effect of CR on the risk of new CV events even in the modern era of MI treatment [19]. However, the population in RCT studies might differ from the general population with chronic CHD. We had a participation rate of 47% in CR, which is higher than the national average of 28% [20]. Non-participation in CR was associated with MACE in analyses adjusted for age and CV risk factors. The effect of CR is thereby likely not only limited to the effect on risk factor control. Factors such as better medical adherence [11] and effect on depressive symptoms [21] might explain some of the additional effect. When adjusting for CV comorbidities, CR non-participation becomes borderline significant (*p* = 0.077).

We found no significant association between higher LDL-cholesterol levels and MACE. This can be explained by the high prescription rate of statins and an average LDL-cholesterol level of 2.1 mmol/L at baseline. It is previously shown that the effect of LDL-cholesterol on cardiac prognosis in chronic CHD is most pronounced in those with levels above 2.6 mmol/L [22]. However, increasing LDL-cholesterol level was significantly associated with recurrent MACE in the subgroup with one coronary event only. These patients are younger and have less comorbidity which may explain the relatively stronger effect on CV prognosis. Not taking a statin was the strongest determinant of recurrent MACE, and remained significant after adjusting for other risk factors and CV comorbidities. Taking statin treatment was also protective of recurrent CV events in EuroAspire IV [7] and REACH [5] registries. Thus, novel strategies to ensure prescription of and long-term adherence to statin therapy seems to be even more important than further LDL-cholesterol reduction in an outpatient CHD population. Muscular side-effects are the major cause of non-adherence/discontinuation of statins [23]. Thus, further research into statin associated muscle symptoms and the identification of a biomarker is of vital importance [23].

Several studies have found an “obesity paradox”, where overweight and moderately obese patients have better prognosis than those with normal weight [24]. Increasing waist circumference was significantly associated with MACE, but not when adjusted for other CV risk factors. In line with our results, EuroAspire IV [2] found a trend towards higher risk of MACE with increasing waist circumference. Diabetes, mainly type 2 (93%) was, associated with MACE in all adjusted analyses except from borderline significance (*p* = 0.061) after adjusting for CV comorbidity. Systolic BP levels were significantly associated with MACE only in crude (1.07, 95% CI 1.00–1.15 per 10 mmHg, *p* = 0.045), but not adjusted analyses, as observed in EuroAspire IV [7]. In line with obesity, the effect of BP on MACE might have been partly modified through other risk factors like diabetes, CV comorbidity and increasing age.

Higher HADS sub-scores of both depression and anxiety were associated with increased risk of MACE in analyses adjusted for coronary risk factors, suggesting that the effect of these factors on MACE risk are not mediated through poor risk factor control alone. A wide range of mechanisms linking psychosocial factors to CHD have been identified, such as proinflammation, endothelial dysfunction and changes in the hypothalamic–pituitary-adrenal and autonomic nervous system [25]. Even though treatment of depression so far has yielded limited and uncertain effect on prognosis [26], depression and other psychosocial factors are important to address as they may act as barriers to both lifestyle changes and treatment adherence [25, 27].

The NOR-COR population was consecutively recruited and the participation rate was high (83%). Socioeconomic status and mortality rate were in line with national data [9]. Another strength of the study is that all MACE have been extracted from the hospital records by experienced cardiologists with only 14 out of 1127 patients being lost to follow-up. Since the hospital records are automatically linked to the Population Registry in Norway, no fatal cases are likely to have been overlooked. The present study has limitations. We may have missed some MACE occurring outside the catchment area of the participating hospitals. However, as hospital discharge reports are normally sent to the local hospital in Norway, the risk is low. By design, patients were included in NOR-COR 2–36 months after the index event, which may introduce a survival bias, as 160 patients had died between time of event and inclusion. These patients may have had even poorer risk factor control or more comorbidity than those included.

Although we have performed a comprehensive evaluation of determinants associated with recurrent MACE, data on additional potentially modifiable factors like fasting blood glucose, the use of metformin and pack years smoked are not available.

## Conclusions

The risk of recurrent CV events remained high in an outpatient coronary population, particularly in the presence of CV comorbidity. Not taking statin therapy, insufficient physical activity, smoking, diabetes, higher depression scores and non-participation in CR were the major preventable and potentially modifiable factors associated with MACE. Potentially preventable clinical and psychosocial factors predicted two out of three MACE, and efforts that target the factors identified may reduce the incidence of recurrent CV events in outpatient coronary populations.

## Supplementary information

**Additional file 1:** Long-term risk of all recurrent cardiovascular events in an outpatient coronary population, estimated by Cox proportional hazard models.

## Data Availability

According to Norwegian legislation, the Norwegian Data Protection Authority and the Committee of Ethics, we are not allowed to share original study data publicly. However, except for anthropometric data, the other essential data by which the conclusions in the article are based will be provided upon reasonable request to the corresponding author.

## Abbreviations

ACEI: Angiotensin converting enzyme inhibitor
ARB: Angiotensin receptor blocker
BP: Blood pressure
CABG: Coronary artery bypass graft operation
CHD: Coronary heart disease
CI: Confidence interval
CR: Cardiac rehabilitation
CRP: C-reactive protein
CV: Cardiovascular
CVD: Cardiovascular disease
eGFR: Estimated glomerular filtration rate
HADS: Hospital anxiety and depression score
LDL: Low density lipoprotein cholesterol
MACE: Major adverse cardiovascular events
MI: Myocardial infarction
PAD: Peripheral artery disease
PAF: Population attributable fraction
PCI: Percutaneous coronary intervention
RCT: Randomized clinical trial
RR: Relative risk
SD: Standard deviation
TIA: Transitory ischemic attacks
