# Supplementary material for: Preventable clinical and psychosocial factors predicted two out of three recurrent cardiovascular events in a coronary population
Source: BMC Cardiovasc Disord. 2020 Feb 5;20:61. doi: 10.1186/s12872-020-01368-6 (PMC7003324; doi:10.1186/s12872-020-01368-6)
Supplement: Supplementary file 1 — Additional file 1: Long-term risk of all recurrent cardiovascular events in an outpatient coronary population, estimated by Cox proportional hazard models. [file 12872_2020_1368_MOESM1_ESM.docx]

| **Additional file 1.** Long-term risk of all recurrent cardiovascular events in an outpatient coronary population, estimated by Cox proportional hazard models | | | | | | | | | | | |
| --- | --- | --- | --- | --- | --- | --- | --- | --- | --- | --- | --- |
|  |  | | | |  | | | | | |  |
|  | **Model 2^a^** | | **Model 3^b^** | | | **Modell 4^c^** | |  |  |  |  |
|  | **RR** | **p-value** | **RR** | **p-value** | | **RR** | **p-value** |  |  |  |  |
| Age per 10 years | 1.22 (1.04, 1.42) | 0.014 | 1.23 (1.04, 1.44) | 0.013 | | 1.07 (0.90, 1.28) | 0.422 |  |  |  |  |
| Male sex | 0.91 (0.65, 1.28) | 0.585 | 0.96 (0.70, 1.31) | 0.790 | | 0.92 (0.69, 1.27) | 0.612 |  |  |  |  |
| Low education^d^ | 1.76. (1.27, 2.45) | 0.001 | 1.65 (1.18, 2.29) | 0.003 | | 1.54 (1.11, 2.16) | 0.010 |  |  |  |  |
| Never smoking | 1 (reference) | | 1 (reference) | | | 1 (reference) | |  |  |  |  |
| Former smoking | 1.43 (0.98, 2.08) | 0.063 | 1.34 (0.92, 1.96) | 0.126 | | 1.25 (0.85, 1.81) | 0.253 |  |  |  |  |
| Current smoking | 1.44 (0.93, 2.22) | 0.100 | 1.30 (0.83, 2.02) | 0.254 | | 1.08 (0.69, 1.69) | 0.736 |  |  |  |  |
| Adequate physical activity^e^ | 1 (reference) | | 1 (reference) | | | 1 (reference) | |  |  |  |  |
| Low physical activity | 1.28 (0.91, 1.80) | 0.155 | 1.25 (0.89, 1.75) | 0.198 | | 1.20 (0.84, 1.70) | 0.316 |  |  |  |  |
| Physical inactivity | 1.90 (1.28, 2.82) | 0.001 | 1.69 (1.08, 2.49) | 0.019 | | 1.59 (1.02, 2.46) | 0.039 |  |  |  |  |
| LDL cholesterol per mmol/L increase | 1.06 (0.87, 1.27) | 0.575 |  |  | |  |  |  |  |  |  |
| Systolic blood pressure per 10 mmHg increase | 1.00 (0.92, 1.08) | 0.936 |  |  | |  |  |  |  |  |  |
| Waist circumference per 10 cm | 1.40 (1.18, 1.66) | <0.001 | 1.14 (0.95, 1.38) | 0.141 | | 1.14 (0.95, 1.38) | 0.166 |  |  |  |  |
| C-reactive protein per mg/L increase | 1.02 (0.98, 1.05) | 0.347 |  |  | |  |  |  |  |  |  |
| Not participating in cardiac rehabilitation | 1.51 (1.11, 2.06) | 0.008 | 1.38 (1.02, 1.87) | 0.037 | | 1.34 (0.97, 1.84) | 0.076 |  |  |  |  |
| Not taking statin | 1.97 (1.28, 3.04) | 0.002 | 1.98 (1.32, 2.97) | 0.001 | | 2.06 (1.36, 3.14) | 0.001 |  |  |  |  |
| Heart failure | 1.59 (1.09, 2.33) | 0.016 | 1.46 (1.00, 2.14) | 0.052 | | 1.31 (0.87, 1.96) | 0.197 |  |  |  |  |
| Peripheral artery disease | 2.08 (1.48, 2.93) | <0.001 | 1.85 (1.28, 2.67) | 0.001 | | 1.82 (1.24, 2.68) | 0.002 |  |  |  |  |
| Stroke or transient ischemic attack | 1.32 (0.80, 2.20) | 0.281 |  |  | |  |  |  |  |  |  |
| Chronic kidney failure (eGFR<60 mL/min/1.73m^2^) | 2.12 (1.43, 3.14) | <0.001 | 1.88 (1.29, 2.74) | 0.001 | | 1.76 (1.19, 2.62) | 0.005 |  |  |  |  |
| HADS Anxiety sum per unit increase | 1.03 (0.99, 1.06) | 0.148 | 1.02 (0.98, 1.06) | 0.258 | | 1.01 (0.98, 1.05) | 0.459 |  |  |  |  |
| HADS Depression sum per unit increase | 1.04 (1.01, 1.08) | 0.022 | 1.02 (0.99, 1.05) | 0.267 | | 1.01 (0.98, 1.05) | 0.435 |  |  |  |  |
| RR: Relative risk; LDL: Low density lipoprotein cholesterol; eGFR: estimated glomerular filtration rate: HADS: Hospital anxiety and depression score  Analyses based on imputed dataset  ^a^Adjusted for age. Analysis is stratified by prior coronary events before the index event or not  ^b^Adjusted for age and coronary risk factors with p-value<0.1 in crude or age adjusted analyses (smoking, LDL cholesterol, physical activity and systolic blood pressure). Analysis is stratified by prior coronary events before the index event or not  ^c^Adjusted for age, coronary risk factors with p-value<0.1 in crude or age adjusted analyses (smoking, LDL cholesterol, physical activity and systolic blood pressure), and cardiovascular comorbidity factors with p-value <0.1 in crude analyses (heart failure, peripheral artery disease and kidney failure). Analysis is stratified by prior coronary events before the index event or not  ^d^ Completion of primary or secondary school only  ^e^Adequate physical activity is defined as moderate physical activity for 30 minutes at least 2-3 times a week, low physical activity is defined as moderate physical activity for less than 30 minutes 2-3 times a week, and physical inactivity is defined as physical activity less than one time a week. | | | | | | | | | | |  |
|  | | | | | | | | | | |  |
|  | | | | | | | | | |  |  |
|  | | | | | | | | | |  |  |
|  | | | | | | | |  | |  |  |
